# Stress and associated risk factors among the elderly: a cross-sectional study from rural area of Thailand

**DOI:** 10.12688/f1000research.17903.2

**Published:** 2020-04-01

**Authors:** Katekaew Seangpraw, Nisarat Auttama, Ramesh Kumar, Ratana Somrongthong, Prakasit Tonchoy, Pitakpong Panta

**Affiliations:** 1 Public Health, School of Medicine, University of Phayao, Phayao, Thailand; 2 School of Nursing,, University of Phayao, Phayao, Thailand; 3 Health System and Policy, Health Services Academy, Islamabad, Pakistan; 4 College of Public Health,, Chulalongkorn University, Thailand, Bangkok, Thailand

**Keywords:** Evaluation factors, stress, elderly rural, risk factors, association

## Abstract

**Background. **Stress is a common mental health problem of the elderly population that affects their quality of life. The objective of this study was to determine the level of stress and associated factors among the elderly living in rural areas of Thailand.

**Methods. **This was a cross-sectional study conducted in two sub-districts of rural Thailand and interviewed 403 elderly persons.  We used simple random sampling technique from a list of registered elderly individuals and conducted face-to-face interviews using a questionnaire. The questionnaire were piloted, validated and pretested beforehand. Multiple linear regression was applied for data analysis. This study was approved by the Ethical Review Committee of the University of Phayao, Thailand.

**Results. **The mean age of the participants was 68 and two thirds (67%) were female. Less than 43% of the participants had moderate, and one third (34%) had high levels of stress. More than half of participants had low level stress management. Stress was significantly associated with alcohol and illness with a predictive power of 3.0% [(R = 0.173, R Square = 0.030) (p<0.05)].

**Conclusion.** We conclude that risk factors such as alcohol and illness affect elderly population living in rural areas of Thailand to a major extent in terms of stress.

## Introduction

Globally, 15% of the elderly population is suffering from mental disorders, and stress is one major mental health problem affecting a sizeable proportion (10–55%) of the elderly population ^1, 2^. The prevalence of stress and anxiety among the elderly population is gradually increasing and expected to reach double in the next one decade ^1^. About one fifth of the world’s aging population lives in Thailand, and their number will increase by 28% in the coming ten years ^3^.

In Thailand, recent surveys have reportedly identified increasing stress and mental health issues. Hospital based data complements this by showing increasing burden of stress and anxiety among the elderly ^4^. Recent research has also suggested that the prevalence of stress is associated with age and the chances of getting this condition has increased in the aging population ^3, 4^.

Secondary data from rural Thailand depicts a high proportion of the elderly population suffering from mental health disorders ^5, 6^. Research suggests that common factors affecting stress among the elderly are family relationship, financial status, social or community environment, physical health and chronic illness ^7– 10^. Nonetheless, the factors associated with stress need further exploration. Hence, we conducted this research to determine the factors affecting stress among the elderly in rural Thailand.

## Methods

### Study design

This was a cross-sectional study carried out between January and April 2017 in Muang District, Phayao Province of Thailand.

### Sample size and selection

The study sample size was calculated by using confidence level of 95%, the coefficient of the error = 5% and population proportion of 0.05 ^11, 12^. Hence, 403 elderly people were interviewed in this study by simple random sampling method from a list of promoting hospitals ^1^ registering elderly patients. Our tool was based on Pender’s theory of health promotion model and stress assessment ^13, 14^. We included male and female elderly persons who were above 60 years old, living in the study area for more than one year and able to communicate. However, those who were admitted with other associated diseases were excluded in this study.

### Data collection

Data collectors were trained and briefed on the study prior to conducting this survey. Face to face interviews of 40 minutes per participant were conducted by adopting the simple random sampling method and the data collectors guided interview. The questionnaire was piloted and pretested on 35 elderly living in outside from the study area with similar settings. Cronbach’s alpha coefficient of the questionnaire was calculated as 0.80 and content validity, a Kuder-Richardson 20 coefficient, was assessed as 0.79. There were three parts of the questionnaire; socio-economic characteristics (age, sex, income, education, marital status etc), the stress assessment test composed of 20 items from Suangprung Stress test-20, and the stress management score (10 items may rating scale on four point Likert scale) ^13, 14^. The stress management section was adapted to the elderly community with questions pertaining to the following; “Feeling desperate in life”, “Cannot stay focused”, “Cannot sleep due to stress or overthinking”, and “Muscle pain in the back or shoulders”. The mean score was calculated from their responses; less stress (0 – 23), moderate stress (24 – 41), high stress (42 – 61) and severe stress (>62) ^14^. The total scores were divided into three levels including low scores (0–30), moderate scores (31–39) and high scores (40–50) ^14^. The questionnaire was piloted and pretested on 35 elderly living in outside from the study area with similar settings. Cronbach’s alpha coefficient of the questionnaire was calculated as 0.80 and content validity, a Kuder-Richardson 20 coefficient, was assessed as 0.79.

### Statistical analysis

Data was analyzed using SPSS Statistics version 20.0. Descriptive and multiple stepwise linear regression analysis was used to investigate the potential predictors of stress among the elderly. The analysis we put in the model 1 is alcohol consumption and the model 2 is present illness like; hypertension, musculoskeletal disorders and diabetes as these were the main variables as per our objectives.. The level of significance for all statistical tests was set at p-value <0.05.

### Ethical statement

All participants were informed regarding the research objectives and procedures of the study and a written informed consent was obtained from all the participants prior to start of the study. All the information of participants was kept confidential. This study was approved by the Ethics Review Committee for research involving human research subjects at the University of Phayao Thailand (No. 2/101/59). Administrative approval was gained from the head of the hospitals before to the study began.

## Results

### Baseline characteristics

The mean age of study participants was 68±7, and more than half (67%) of participants were women. About half (50%) of the participants were single, having no education (62%), received monthly income less than 100 US$ (73%). Present illness was defined as having a chronic illness at time of sampling (Hypertension, musculoskeletal disease and hypertension). Around two thirds (63%) of the respondents reported a present illness; hypertension (52%), musculoskeletal disorders (29%), and diabetes mellitus (19%). About two thirds (69%) of participants lived with family members. Almost half of study participants consumed alcohol (45%) and 27% smoked cigarettes ( Table 1).

**Table 1.** Socio-demographic characteristics of elderly (n=403).

Socio-economic factors		
Variables	Categories	N (%)
*Age (min= 60, max= 89,* *mean= 68.04, S.D= 7)*	60-79	376 (93.3)
	≥ 80	27 (6.7)
*Gender*	Male	132 (32.8)
	Female	271 (67.2)
*Education*	No education	250 (62.0)
	Higher than primary school	153 (38.0)
*Marital status*	Single (widowed/divorced/separate)	205 (50.9)
	Married	198 (49.1)
*Income* *(per month US$)*	≤100	294 (73.0)
	≥101	109 (27.0)
*Present illness among elderly (252 out of 403)* *Hypertension* *Musculoskeletal diseases* *Diabetes mellitus*		252 (62.5) 131 (52.0) 73 (29.0) 48 (19.0)
*Living arrangement*	Living alone	125 (31.0)
	Living with family (Spouse and / or children)	278 (69.0)
*Alcohol consumption*	Never consumed	220 (54.6)
	Has consumed	183 (45.4)
*Smoking status*	Non-smoker	293 (72.7)
	Smoker	110 (27.3)

Table 2 shows stress levels among elderly people during the last three months as calculated using the Suangprung Stress test-20 stress assessment test. Almost half of these participants experienced a moderate level of stress (43%). Around 34% experienced a high level of stress and 18% had a low level of stress.

**Table 2.** Number and percentage of stress level among elderly as calculated using the Suangprung Stress test-20 stress assessment test (n=403).

Stress	n	%
Low level (0–23 scores)	74	18.3
Moderate level (24–41 scores)	172	42.7
High level (42–61 scores)	137	34.0
Severe level (≥62 scores)	20	5.0

In term of stress management during the last three months, the results showed that more than half of participants had a low level of stress management (59%), followed by moderate and high levels of stress management (33% and 8%, respectively) ( Table 3).

**Table 3.** Number and percentage of stress management level among elderly (n=403).

Stress management	n	%
Low level (0–30 scores)	238	59.1
Moderate level (31–39 scores)	133	33.0
High level (40–50 scores)	32	7.9

### Relationship between personal factors and stress among elderly people

There was statistically significant relationship between alcohol consumption and present illness with stress levels, as calculated using the Suangprung Stress test-20 stress assessment test ( Table 4).

**Table 4.** Multiple linear regression analysis of alcohol consumption (model 1) and present illness (model 2).

Source Variance	*df*	*SS*	*MS*	*F*	*p-value*
**Model 1**					
Regression	2	1021.555	1021.555	8.155	<.01
Residual	401	125.262	125.262		
Total	403	51251.752			
**Model 2**					
Regression	2	1526.017	763.008	6.138	<.01
Residual	401	49725.735	124.314		
Total	403	51251.752			

*Model* 1 *R =0.141, R2 Square = 0.020, S.E = 11.192, n =403, Model 2 R = 0.173, R2 Square = 0.030, S.E = 11.149, n =403*

The stress scores is 2.95 points higher ( *b* coefficient, Table 5) than the elderly who drink alcohol than those who did not use alcohol. This indicates use of alcohol among elderly is positively associated with their current illness, likely due to their perception that the alcohol will help with mental relaxation. In contrast, if the elderly continue consuming alcohol, the present illness will result in increased stress for the participants. ( Table 5).

**Table 5.** Constant and regression coefficient of alcohol consumption and present illness.

Variables	*b*	*SE.*	*Beta*	*t*	*p-value*
**Model 1**					
Constant	38.573	0.755	-	51.119	<0.001
Alcohol consumption	3.198	1.120	.141	2.856	<0.001
**Model 2**					
Constant	37.230	1.005	-	37.063	<0.001
Alcohol consumption	2.952	1.122	0.130	2.630	<0.01
Present illness	2.325	1.154	-100	2.014	<0.05

Present illness: no (0), yes (1); Alcohol consumption: no (0), yes (1). * = significant p-value

## Discussion

In the present study, the majority of elderly people had moderate and high levels of stress during the last three months. This level of stress among the elderly population could negatively affect their health and well-being ^7, 15^. Other studies elsewhere have shown stress’s drafting effects, indicting that stress would directly effect mental and physical status among the elderly ^3, 15^. Our findings are consistent with a previous study ^15^. Further according to the wear and tear theory, when the elderly population are experiencing poor physical and mental health, they would more likely to develop anxiety ^16, 17^. Chronic diseases and economic problems are the major causes of stress among the elderly. Moreover, long term stress and anxiety can also lead to depression and suicidal tendencies among the elderly ^9, 17^. Studies in South Korea and Denmark found that higher levels of perceived stress were associated with higher mortality ^18– 20^.

Those elderly participants had a low level of stress management were living with their grandchildren. Hence, the elderly living in joint family and took responsibilities including household, grandchildren and financial support to the family found low level of stress as compare to those who live alone ^3, 15^. However, few studies shows that these responsibilities would tend to develop stress and anxiety among elderly. Contrary on other hand study showing emotional attachment was a major contributing factor leading to mental health problems among the elderly ^17^.

In the present study, the two main factors associated with stress among the elderly were alcohol consumption and present illness. Stressed elderly individuals usually prefer alcohol to achieve mental relaxation ^21^. Research shows that negative feelings including stress, disappointment, hatred and unsuccessful can lead to drinking behavior ^21^. Previous research show a strong positive correlation between stress and drinking alcohol, especially among the elderly population ^22^. Moreover, present illness is a predictive power of stress among the elderly where current illness could influence daily life activities. Mental health problems and living in a stressful condition could impact their physical health, sleeping and quality of life ^23^. The literature compliments our findings that chronic illnesses might affect the level of stress among elderly people ^24, 25^. A study performed on elderly people living with hypertension showed that there was a statistically significant relationship between chronic illness and stress ^26^. Our findings are also consistent with a studies on elderly people with diabetes leading to anxiety and stress, ultimately developing depression among this aging population ^27^.

## Conclusion

This study provides an understanding of current mental health situations and factors affecting stress, such as alcohol consumption and illness, of elderly people living in rural communities of Thailand. Non-communicable diseases including hypertension, diabetes, and musculoskeletal disorders are the leading factors shown to develop stress and anxiety.

## Data availability

### Underlying data

Open Science Framework: Stress and associated risk factors among the elderly: a cross sectional study from rural Thailand study, https://www.doi.org/10.17605/OSF.IO/XVKSW ^28^

This project contains the following underlying data:

- Data dictionary for statistic analysis plan.doc (data dictionary)
- Update Data set.xls (Participant data)

### Extended data

Open Science Framework: Stress and associated risk factors among the elderly: a cross sectional study from rural Thailand study, https://www.doi.org/10.17605/OSF.IO/XVKSW ^28^ ****

This project contains the following extended data:

- questionnaire_stress.doc (study questionnaire)

Data are available under the terms of the Creative Commons Zero “No rights reserved” data waiver (CC0 1.0 Public domain dedication).

## Footnotes

^1^Hospitals in Thailand are operated by both the public and private sector to provide medical services for prevention, cure and rehabilitation of patients with medical and health-related conditions
