# Supplementary material for: Stress and associated risk factors among the elderly: a cross-sectional study from rural area of Thailand
Source: F1000Res. 2020 Apr 1;8:655. Originally published 2019 May 13. [Version 2] doi: 10.12688/f1000research.17903.2 (PMC7122445; doi:10.12688/f1000research.17903.2)
Supplement: Peer review history

# Peer review: Reviewer response for version 2

**DOI:** 10.5256/f1000research.25465.r61907

**Published:** 2020-04-02

**Authors:** Kraichat Tantrakarnapa

**Affiliations:** 1 Department of Social and Environmental Medicine, Faculty of Tropical Medicine, Mahidol University, Bangkok, Thailand

It seems to be fine as the authors responded to the comments.

I confirm that I have read this submission and believe that I have an appropriate level of expertise to confirm that it is of an acceptable scientific standard.

# Peer review: Reviewer response for version 2

**DOI:** 10.5256/f1000research.25465.r61906

**Published:** 2020-04-01

**Authors:** Thant Zaw Lwin

**Affiliations:** 1 Department of Public Health, Union Solidarity and Development Party, Nay Pyi Taw, Myanmar

I approve this article.

I confirm that I have read this submission and believe that I have an appropriate level of expertise to confirm that it is of an acceptable scientific standard.

# Peer review: Reviewer response for version 1

**DOI:** 10.5256/f1000research.19579.r53600

**Published:** 2019-09-24

**Authors:** Kraichat Tantrakarnapa

**Affiliations:** 1 Department of Social and Environmental Medicine, Faculty of Tropical Medicine, Mahidol University, Bangkok, Thailand

**Research Title:**

1. It seems to be clear. However, it was conducted only in sub-districts in one province of Thailand. The title is a big one; how can this study represent the rural area of Thailand? There were 7,435 sub-districts in 2018. Only two sub-districts were selected as the representative of the rural area.

**Introduction:**

1. “Hence, we conducted this research to determine the factors affecting stress among the elderly in rural Thailand.” It was indicated in the manuscript. The title and the significance of this study focus on the overview of Thailand, whereas the study areas examined were only 2 sub-districts and Muang District (city). Can they be representatives for rural areas? As indicated in Research methodology.

**Methods:**

1. “The study sample size was calculated by using a confidence level of 95%, the coefficient of the error = 5% and the population proportion of 0.05.” Authors got 403 sample size. What is the formula for sample size calculation?
2. “Hence, 403 elderly people were interviewed in this study by simple random sampling method from a list of promoting hospitals registering elderly patients”. Do the researchers collect only elderly patients? Can they be the representatives for elderly people there?
3. Are the subjects Thai nationality? In this province, there might be some migrants who lived in this province for more than one year and they can communicate in Thai?
4. “Face to face interviews of 40 minutes per participant were conducted”. Is it a digit for interviewing time? Time is used for controlling the interview.
5. Authors indicated that the tools were tested prior to usage. Is IOC used for testing the content validity? For example, “Cannot sleep due to stress or overthinking”, the question was asked directly to the point of stress, is it biased?
6. Cronbach’s alpha coefficient of the questionnaire was calculated as 0.80. It might be the total score of a questionnaire?
7. “Kuder-Richardson 20 coefficient, was assessed as 0.79.” Please clarify the meaning of this statement.
8. Data were analyzed using SPSS Statistics version 20.0. Is this a licensed software?
9. Model 2 is a present illness like; hypertension, musculoskeletal disorders and diabetes. Does this variable count for “existing illness” and “no existing illness” or consider the number of existed diseases for a regression analysis?
10. Administrative approval was gained from the head of the hospitals before the study began. Does this study focus on the elderly in the hospital?

**Results:**

1. Having no education, could the authors use another word replacing “no education”?
2. Income (per month US$).
3. The researchers did it during 2017, at that time the Thai Baht value is different from the current situation (please specify the exchange rate).
4. Smoking and alcohol drinking status, the authors classified only 2 categories “Yes” and “No” at the interviewing time. The experience of consumption or smoking were not considered.  Some just quit or stop smoking or drinking, are they influencing the stress?
5. In term of stress management as indicated in the manuscript. Please describe more details of this issue. What are they? For example; low level of management, what does it mean?
6. *“*This indicates the use of alcohol among the elderly is positively associated with their current illness, likely due to their perception that the alcohol will help with mental relaxation. *”* Is there any scientific information supporting this statement?
7. For the results indicated in Table 5, are there any perception variables used in the model? Because of drinking, are they facing the stress or they have stress then they decided to release it by drinking?

**Conclusion**:

1. It is not clear for the conclusion and recommendation is not available there.

I confirm that I have read this submission and believe that I have an appropriate level of expertise to confirm that it is of an acceptable scientific standard, however I have significant reservations, as outlined above.

# Response

**Published:** 2020-01-10

**Authors:** Ramesh Kumar

**Affiliations:** Health Services Academy, Pakistan

Title and introduction: we have selected two sub-rural districts in the study randomly that was truly representative of this rural population.

Methods:

1. We used the confidence level of 95%, the coefficient of the error = 5% and population proportion of 0.05. 

2. Yes, only elderly population were included in this study.

3. Yes only Thai national were included.

4. Yes we agree.

5. No, we have asked only those questions were included in the tool.

6. Yes this was Cronbach’s alpha coefficient of the questionnaire was calculated as 0.80.

7. Kuder-Richardson 20 coefficient, was assessed as 0.79 was used to check validity of the questionnaire.

8. Yes that was licence software.

9. Only existing illness.

10. Administrative approval from hospital was sought to include elderly population in the study from the registered record available with them.

Results: 

1. No.

2. Yes converted thai bath in the US$

3.Conversion rate was taken at that time.

4. Included those who were actively smoking or not

5. We have categorised the stress level and measured accordingly.

6. This information supported in the discussion part.

7. Yes.

Conclusion: We have achieved our objectives and concluded accordingly and did not included the recommendation as per the Journal's criteria.

# Peer review: Reviewer response for version 1

**DOI:** 10.5256/f1000research.19579.r48413

**Published:** 2019-05-29

**Authors:** Thant Zaw Lwin

**Affiliations:** 1 Department of Public Health, Union Solidarity and Development Party, Nay Pyi Taw, Myanmar

**In Abstract**

 There was no recommendation in conclusion section, or is there a word limit for the abstract? In the keywords: there was no mention of "evaluation factors" in the abstract, and what do you mean by "Elderly rural" or "association"?

**In Introduction**

 2nd paragraph; "In Thailand, recent surveys …" and last line in that paragraph "Recent research …", these two lines should shift to the conclusion section.

**In Methods**

 What about the sampling procedure?

**In Data Collection**

 Face to face interviews of 40 minutes and the results showed that 62% have no education, is it possible that it took too much time to get the right answers. What about their responses?

 This survey was pre-tested and validated outside of the study, so what are the operational definitions from this?

**In Results**

 67% of participants were women (meaning that 33% were men). But in Table 1, alcohol consumption was 45.4%. Does this mean all the men has alcohol consumption and few of elderly women has alcohol consumption?

 50% of the participants were single but in the discussion, more than half of the elderly participants had a low level of stress management and were living with their grandchildren. And in the results about two thirds (69%) of participants lived with family members. What about these two connections?

 In Table 4, why the sample is 402 and why not 403?

**In Conclusion**

 No recommendation or other relevant evidence for association?

 How about recommendations for the next study?

**In References**

 No. 28 reference is where citations in manuscript?

**Overall Conclusions**

 Please answer my questions and correct for some facts. Its just minor corrections.

I confirm that I have read this submission and believe that I have an appropriate level of expertise to confirm that it is of an acceptable scientific standard, however I have significant reservations, as outlined above.

# Response

**Published:** 2020-01-10

**Authors:** Ramesh Kumar

**Affiliations:** Health Services Academy, Pakistan

Abstract: Recommendations are included in the main paper, here we can not include due to word limit. Evaluating factors are not included in our objectives, elderly rural are the key words.

Introduction: Recent does not mean about this research, we are talking about other recent research to triangulate with our work. Hence it is possible to move in conclusion section.

Methods: Simple random sampling method was adopted. Face to face interview was guided by the data collectors and findings were included in the result section.The questionnaire was piloted and pretested on 35 elderly living in outside from the study area with similar settings. Cronbach’s alpha coefficient of the questionnaire was calculated as 0.80 and content validity, a Kuder-Richardson 20 coefficient, was assessed as 0.79.

Results: Table 1 findings are truly representing our findings. We have mentioned 50% like nearly half in the results. Typo error will be corrected by 403 in table 4.

Conclusion: Recommendations are not included as per the Journal's criteria.

References: 28 will be added with 27 in the list
